# Severe sleep disturbance is associated with executive function impairment in patients with first-episode, treatment-naïve major depressive disorders

**DOI:** 10.1186/s12888-021-03194-2

**Published:** 2021-04-19

**Authors:** Feihuan Cui, Qi Liu, Xiaozhen Lv, Rainer Leonhart, Hongjun Tian, Jing Wei, Kerang Zhang, Gang Zhu, Qiaoling Chen, Gang Wang, Xueyi Wang, Nan Zhang, Yu Huang, Tianmei Si, Xin Yu

**Affiliations:** 1 Department of Psychology, Beijing Anzhen Hospital, Capital Medical University, Beijing, China; 2 Peking University Sixth Hospital, Peking University Institute of Mental Health, No. 51 Huayuanbei Road, Beijing, 100191 China; 3 NHC Key Laboratory of Mental Health (Peking University), National Clinical Research Center for Mental Disorders (Peking University Sixth Hospital), Beijing, China; 4 Department of Social Psychology and Methodology, Institute for Psychology, University of Freiburg, Freiburg, Germany; 5 Nankai University Affiliated Anding Hospital, Tianjin Mental Health Center, Tianjin, China; 6 Department of Psychological Medicine, Peking Union Medical College Hospital, Beijing, China; 7 Department of Psychiatry, First Hospital of Shanxi Medical University, Taiyuan, Shanxi China; 8 Department of Psychiatry, The First Affiliated Hospital of China Medical University, Shenyang, Liaoning China; 9 Department of Psychiatry, Dalian Seventh People’s Hospital, Dalian, China; 10 Beijing Anding Hospital, Capital Medical University, Beijing, China; 11 Department of Psychiatry, The First Hospital of Hebei Medical University, Mental Health Institute of Hebei Medical University, Shijiazhuang, China; 12 Department of Neurology, Tianjin Medical University General Hospital, Tianjin, China; 13 National Engineering Research Center for Software Engineering, Peking University, Beijing, China

**Keywords:** Major depressive disorder, Cognitive functioning, Sleep disturbance, Executive functioning

## Abstract

**Background:**

Sleep disturbance and executive function impairment are common in patients with major depressive disorder (MDD), though the relationship between the two remains unclear. We investigated this association in first-episode, treatment-naïve patients with MDD.

**Methods:**

We analyzed data from 242 patients with MDD. We divided the patients into 2 groups based on sleep disturbance severity and compared the executive function odds ratios between the groups.

**Results:**

A total of 121 pairs of patients were matched (age 39.4 ± 10.1, 70.2% female). After propensity score matching, the odds ratios for cognitive impairment in patients with MDD and severe sleep disturbance were 1.922 (1.068–3.459, *P* = 0.029, *q* = 0.044) in executive functioning; 2.023 (1.211–3.379, *P* = 0.007, *q* = 0.021) in executive shifting.

**Conclusions:**

Sleep disturbance is associated with executive functioning impairment in first-episode, treatment-naïve patients with MDD. Severe sleep disturbance can be a marker and aid in recognizing executive function impairment in patients with first-episode treatment-naïve MDD. Severe sleep disturbance can be a potential modifiable factor to improve executive function in MDD, as well as an effective measurement to improve cognition for sleep symptom management that should be enforced at initial treatment of first-episode MDD. Further study is required to confirm our results.

**Trial registration:**

ClinicalTrials.gov: NCT02023567; registration date: December 2013.

**Supplementary Information:**

The online version contains supplementary material available at 10.1186/s12888-021-03194-2.

## Background

Major depressive disorder (MDD) is a disabling disease with significant social and economic consequences, including decreased work productivity and poor psychosocial outcomes [1, 2]. MDD has been associated with cognitive impairment in several domains, including attention, memory, executive functioning, and information processing speed [3], and approximately 90% of patients with MDD complain about impaired cognition [4].

Patients with first-episode MDD can suffer from impaired cognition [5]. Cognitive dysfunction especially executive function impairment is associated with a high risk of relapse and a low rate of remission [6, 7], increasing the risk of treatment-resistant MDD, so it is imperative to intervene early during the course of the first episode of MDD. Executive impairment has 3 dimensions including inhibition (defined as suppressing or avoiding an automatic response), shifting (defined as switching between task sets and response rules), and work memory (defined as actively maintaining or manipulating information across a short delay, which can be further divided into verbal and visuospatial components) [8], and has been identified as one of the main impaired cognitive domains in patients with MDD [8]. Deficits in executive functioning have been associated with pathophysiology in the prefrontal cortex-subcortical brain circuitry in MDD patients [9].

Sleep disturbances are common complaints in patients who are depressed [9], and they, in turn, increase the risk of developing a depressive disorder [10–12]. Sleep disturbances are manifest in different ways, including difficulty falling asleep, frequent nocturnal awakenings, and waking early in the morning, unable to go back to sleep, resulting in sleep fragmentation and poor sleep efficiency. Studies about sleep electroencephalograms in MDD patients have found disinhibition of rapid eye movement (REM) sleep- a decrease in REM sleep latency and an increase in REM sleep duration [13]. The prefrontal cortex (PFC) is deactivated and decoupled from posterior associative regions during REM sleep [14].

Therefore, potential complex relationships are existed among sleep disturbance, depressive disorder, and executive function impairment. Executive function impairment has been reported in old insomnia patients [15]. Functional alterations in the prefrontal cortex, cingulate gyrus, and subcortical regions, combined with deficits in executive functioning, have been observed in patients with sleep disorders [16]. A recent meta-analysis concluded that insomnia has a profound effect on a wide range of cognitive domains, including manipulation in working memory, problem-solving, and perceptual functions [17]. Insomnia has been reported to relate to impairment in executive function and visual-motor processing speed in MDD patients [18]. However, the result is the opposite in the individuals with chronic fatigue syndrome, and the sleep symptoms are not related to impaired cognitive function [19]. The previous studies mainly selected health individuals as the control group with small sample sizes.

Researchers have found several risk factors for executive impairment in patients with MDD, including age [20], age of onset [21], depression severity and duration, comorbid physical disease, episode frequency [1], childhood trauma [22, 23], and MDD subtype [24]. Most studies have focused on recurrent depressive disorders or cognitive impairment in late-onset depression [25]. Some risk factors are unmodifiable, such as age, and others are potentially modifiable, such as MDD severity. Though previous studies have explored the negative consequences of sleep disturbances in patients with MDD or executive function impairment [11, 26, 27], it is still not known about the role, if any, of sleep disturbances play in executive function impairment in patients with MDD.

Previous studies have included patients with both first-episode and recurrent depressive disorders, and the influence of antidepressants has not been clarified. So, we hypothesize that severe sleep disturbance is associated with poorer executive function and its dimensions in MDD patients than the patients with normal or up to moderate sleep disturbance. If so, then treating sleep disturbances may be an option in reducing executive function impairment in a clinical setting. Here we report on the relationship between sleep disturbances and executive function impairment in the setting of a clinical trial.

## Methods

### Participants

Between December 2013 and December 2016, researchers recruited Chinese adults to participate in the multicenter, multistage, prospective study Objective Diagnostic Indicators and Individualized Drug Intervention of Major Depressive Disorder (OIMDD), project No. 2013CB531305. We conducted a secondary, cross-sectional analysis using the baseline data from OIMDD for participants diagnosed with MDD. Nine hospitals in China participated in the study. All participants provided written consent, and the study protocol was approved by the research ethics board of the institution where it was performed.

Study participants included patients between the ages of 18 and 65 years who had been diagnosed with their first episode of MDD, did not receive any antidepressant treatment in the acute phase of the disease, and had a total score of ≥14 on the 17-item Hamilton Rating Scale for Depression (HAMD-17) [28]. Patients were excluded if they had severe somatic diseases, such as severe heart disease, malignant tumors, or a history of epilepsy. Pregnant or lactating women were also excluded [29].

### Depression diagnosis and clinical assessment

Using criteria from the Diagnostic and Statistical Manual of Mental Disorders, Fourth Edition (DSM-IV), psychiatrists made a diagnosis of MDD and then conducted structured clinical interviews with the prospective participants using the Mini-International Neuropsychiatric Interview (MINI), Chinese Version 5.0.0 [30]. After the interviews, HAMD-17 was used to assess depressive symptoms, evaluating functioning in 5 subscales: cognitive impairment, retardation, anxiety or somatization, sleep disturbance, and weight change [31]. Sleep disturbance severity was assessed by evaluating the following metrics on the HAMD-17 scale: item 4, difficulty falling asleep; item 5, waking in the middle of the night for any reason except to void; and item 6, waking early in the morning and unable to go back to sleep. Each item could be rated from 0 (no difficulty) to 2 (nightly difficulty), with a total possible score of 6. A sleep subscale score > 4 was defined as a severe sleep disturbance, and patients in this category were assigned into Group 1; the remainder, were assigned into Group 2. The Hamilton Anxiety Rating Scale (HAMA) was used to assess 2 areas of somatic anxiety: muscular (pains and aches, twitching, stiffness, myoclonic jerks, grinding of teeth, unsteady voice, and increased muscular tone) and sensory (tinnitus, blurring of vision, hot and cold flushes, feelings of weakness, and pricking sensation) [31, 32].

### Executive function tests

A battery of cognitive tests was performed to evaluate executive functions including dimensions of executive inhibition that was assessed by the Stroop Color Word Test (SCWT), and executive-shifting that was assessed by color line II. We selected the cognitive test battery in our study from the MCCB (MATRICS Consensus Cognitive Battery) of the MATRICS (the Measurement and Treatment Research to Improve Cognition in Schizophrenia) study. The MCCB has been widely used to evaluate the cognitive function of patients with mental disorders. The test scores for each domain were transferred into global deficit scores (GDS), which were adjusted for age, sex, and education level. A global deficit score ≥ 0.5 was defined as cognitive impairment [33, 34].

### Confounding covariables

In addition to gathering basic demographic data—marital status, living situation (whether the subject lives alone or with others), religious affiliation, work status, the type of work (whether it requires mental labor, physical labor, or both), independence (degree to which the subject depends on someone else for life’s basics), and body mass index (BMI), we obtained information on potential confounding covariables that could alter a person’s risk for cognitive impairment. Some comorbid conditions are known to increase the risk of cognitive impairment, including cardiovascular disease and diabetes. Other suspected risk factors include duration of disorder, severity of depression and/or anxiety, family history of psychiatric disorders, history of alcohol abuse, smoking history, and childhood trauma [1].

We collected previous disease history at baseline, including hyperlipidemia, hypertension, and diabetes. Diabetes was defined as a fasting blood glucose (FBG) level ≥ 126 mg/dL (7.0 mmol/L), oral glucose tolerance test ≥200 mg/dL (11.1 mmol/L), HbA1c ≥48 mmol/L (6.5%), or a history of diabetes mellitus. Hyperlipidemia was defined as having total cholesterol (TC) ≥5.17 mmol/L, triglycerides (TG) ≥1.7 mmol/L, low-density lipoprotein (LDL) cholesterol ≥3.37 mmol/L, or a history of hyperlipidemia. Social demographic variables were assessed using questionnaires that asked about age, sex, marital status, occupation, alcohol use, and smoking status. Body mass index was calculated as weight in kilograms divided by squared height in meters (kg/m^2^). BMI was divided into low weight (BMI < 18.5), normal weight (BMI ≥18.5 to < 24), overweight (BMI > 24 to ≤28), and obesity (BMI > 28).

Childhood trauma was assessed using the childhood trauma questionnaire (CTQ), which evaluates the subject’s experience of abuse and/or neglect before the age of 18 years. The CTQ includes domains of emotional, physical, and sexual abuse, as well as of emotional and physical neglect. Items are rated from 1 (“never true”) to 5 (“very often true”) according to the frequency with which each event occurred in childhood. We considered the history of trauma as a dichotomic variable (yes/no) if the person rated items as moderate or severe according to the subscale cutoff criteria in at least one type of trauma [1, 35].

### Statistical analyses

Summary statistics were presented as mean ± standard deviation for variables that conformed to a normal distribution, as medians and quartile for data did not conform to a normal distribution, and as percentages for categorical variables. To make the 2 groups more comparable, we performed propensity score matching. Propensity scores were estimated using a logistic regression model that contained known or suspected covariates that were unbalanced between the 2 groups. To perform the propensity score matching, we included the type of work, marital status, religion, alcohol use, and HAMD-17 weight subscale. Because the independent-variable global deficit scores are functions converted from standard scores corrected by age, sex, and education, we did not include these variables in the propensity score matching. Subjects were matched 1:1 without replacement, using a 0.00 caliper width. Effect size was calculated to estimate the balance of the baseline data between the 2 groups. Cohen d was calculated using a t test and φ(phi) or φc (Cramer’s phi) was calculated using the chi-square test. Effect size > 0.20 was considered to be an imbalance between the 2 groups. For multiple hypothesis tests with same data settings, to reduce false positive, correction was analyzed with the method of Benjamini and Hochberg, and corrected *p* value (*q* value) was calculated.

Statistical analyses were conducted using SPSS 25.0 (SPSS Inc., Chicago, Illinois, USA) and version R 3.3.3 (Foundation for Statistical Computing, Vienna, Austria). The R package of MatchIt was used for the propensity score analysis. Logistic regression analysis was used to estimate the odds ratios for the global deficit scores for each cognitive domain and their corresponding 95% confidence intervals (CIs).

## Results

Of the 296 patients included in our analysis, 121 had symptoms of severe sleep disturbance. Chi-square analysis and nonparametric test of the demographic and clinical characteristics between the 2 groups revealed significant differences in the groups’ social demographics (see Table 1), including type of work (10.924, *P =* 0.004), marital status (17.072, *P* < 0.001), living situation (19.919, *P* < 0.001*)*, religion (4.457, *P* = 0.035), alcohol use (14.284, *P* = 0.001), and HAMD weight subscale (*P* = 0.001). Because the 2 groups were so heterogeneous, we performed propensity score matching and the variables above were included into matching, after which the baseline characteristics of the 2 groups were highly comparable and we were able to successfully match 121 pairs of participants (average age 39.42 ± 10.07, 70.2% female). No differences in the other variables were observed between the 2 groups, with the exception of age (Table 2).

**Table 1:** Demographic and clinical characteristics of study participants before matching

	Group 1 (121)	Group 2 (175)	Effect Size	t, z, or Chi-square	*P* value
Age	41.8 ± 9.2	35.1 ± 10.6	0.675	−5.600 ^a^	< 0.001
Education years (year)	11.9 ± 4.0	13.1 ± 3.7	0.311	2.588 ^a^	0.010
Sex (male) (%)	39 (32.2)	52 (29.7)	0.027	0.213 ^b^	0.645
Occupation (%)					
Full-time or part-time	83 (68.6)	127 (72.6)			
Unemployed or retired	38 (31.4)	48 (27.4)	0.043	0.549 ^b^	0.459
Nature of work (%)					
Mental talents labor	58 (47.9)	117 (66.9)			
Physical labor	39 (32.2)	33 (18.9)			
Both	24 (19.9)	25 (14.3)	0.192	10.924 ^b^	0.004
Marital status (%)					
Married or remarried	101 (83.5)	107 (61.1)			
Divorced or widowed or single	20 (16.5)	68 (38.9)	0.240	17.072 ^b^	< 0.001
Living situation (%)					
Living alone	12 (9.9)	15 (8.6)			
With parents or spouse	31 (25.6)	66 (37.7)			
With spouse and child	9 (7.4)	35 (20.0)			
Other	69 (57.1)	59 (33.7)	0.259	19.919 ^b^	< 0.001
Independence (%)					
Independent	115 (95.0)	156 (89.1)			
Need assistance	6 (5.0)	19 (10.9)	0.104	3.219 ^b^	0.073
Childhood trauma (%)					
Yes	39 (32.2)	71 (40.6)	0.085	2.131 ^b^	0.144
Religion (%)					
No	117 (96.7)	158 (90.3)	0.123	4.457 ^b^	0.035
Smoking history (%)					
Never	89 (73.6)	136 (77.7)			
Quit smoking	11 (9.0)	7 (4.0)			
Smoking now	21 (17.4)	32 (18.3)	0.105	3.246 ^b^	0.197
Alcohol use (%)					
Never	89 (73.6)	106 (60.6)			
Quit drinking	10 (8.3)	5 (2.8)			
Drinking now	22 (18.1)	64 (36.6)	0.220	14.284 ^c^	0.001
Hypertension (%)					
Yes	14 (11.6)	10 (5.7)	0.105	3.293 ^b^	0.070
Diabetes (%)					
Yes	7 (5.8)	4 (2.3)	0.091	2.448 ^c^	0.131
Hyperlipidemia (%)					
Yes	9 (7.4)	18 (10.3)	0.049	0.700 ^b^	0.403
First degree relatives (%)					
Yes	20 (16.5)	33 (18.9)	0.030	0.264 ^b^	0.608
Duration of MDD (months)	6.0 (2.0, 12.0)	6.0 (3.0, 21.0)		−1.513 ^d^	0.130
BMI (%)			0.144	6.157 ^b^	0.104
Low weight	11 (9.1)	18 (10.3)			
Normal	59 (48.8)	107 (61.1)			
Overweight	41 (33.8)	42 (24.0)			
Obesity	10 (8.3)	8 (4.6)			
HAMD anxiety subscale	5.8 ± 2.2	6.1 ± 1.8	0.149	0.972 ^a^	0.332
HAMD weight subscale	0.0 (0.0, 2.0)	0.0 (0.0, 1.0)		−3.422 ^d^	0.001
HAMD cognition subscale	3.0 ± 1.9	3.1 ± 1.6	0.057	0.493 ^a^	0.623
HAMD retard subscale	6.8 ± 1.7	6.5 ± 1.8	0.171	− 1.534 ^a^	0.126
HAMA somatic anxiety	13.4 ± 4.1	13.2 ± 3.7	0.051	− 0.544 ^a^	0.687
HAMA psychological anxiety	6.9 ± 4.3	7.5 ± 4.2	0.141	1.182 ^a^	0.238

*G1* Group 1, patients with severe sleep disturbance, *G2* Group 2, patients without severe sleep disturbance

^a^ Independent sample t-test; ^b^ chi-square test; ^c^ Fisher’s exact test; ^d^ nonparametric test

**Table 2:** Demographic and clinical characteristics after matching

	Group 1 (121)	Group 2 (121)	Effect Size	t, z, or Chi-square	*P* Value
Age	41.8 ± 9.2	37.1 ± 10.4	0.478	−3.699 ^a^	< 0.001
Education years	11.9 ± 4.0	12.7 ± 3.8	0.205	1.588 ^a^	0.114
Sex (male) (%)	39 (32.2)	33 (27.3)	0.054	0.712 ^b^	0.399
Occupation (%)					
Full-time or part-time	83 (68.6)	86 (71.1)			
Unemployed or retired	38 (31.4)	35 (28.9)	0.027	0.177 ^b^	0.674
Nature of work (%)					
Mental talents labor	58 (48.0)	75 (62.0)			
Physical labor	39 (32.2)	28 (23.1)			
Both	24 (19.8)	18 (14.9)	0.141	4.836 ^b^	0.089
Marital status (%)					
Married or remarried	101 (83.5)	89 (73.6)			
Divorced, widowed, or single	20 (16.5)	32 (26.4)	0.121	3.527 ^b^	0.060
Living situation (%)					
Living alone	12 (9.9)	9 (7.4)			
With parents or spouse	31 (25.6)	42 (34.7)			
With spouse and child	9 (7.4)	17 (14.1)			
Other	69 (57.1)	53 (43.8)	0.166	6.646 ^b^	0.084
Independence (%)					
Independent	115 (95.0)	112 (92.6)			
Need assistance	6 (5.0)	9 (7.4)	0.051	0.640 ^b^	0.424
Childhood trauma (%)					
Yes	39 (32.2)	46 (38.0)	0.061	0.889 ^b^	0.346
Religion (%)					
No	117 (96.7)	114 (94.2)	0.061	0.857 ^b^	0.355
Smoking (%)					
Never	89 (73.6)	96 (79.3)			
Quit smoking	11 (9.0)	5 (4.1)			
Smoking now	21 (17.4)	20 (16.5)	0.102	2.539 ^c^	0.281
Alcohol use (%)					
Never	89 (73.6)	84 (69.4)			
Quit drinking	10 (8.2)	4 (3.3)			
Drinking now	22 (18.2)	33 (27.3)	0.143	4.916 ^c^	0.086
Hypertension (%)					
Yes	14 (11.6)	9 (7.4)	0.105	1.201 ^b^	0.381
Diabetes (%)					
Yes	7 (5.8)	3 (2.5)	0.091	1.669 ^c^	0.333
Hyperlipidemia (%)					
Yes	9 (7.4)	13 (10.7)	0.049	0.800 ^b^	0.371
First degree of relatives (%)					
Yes	20 (16.5)	20 (16.5)	0.030	0.000 ^b^	1.000
Duration of MDD (months)	6.0 (2.0, 12.0)	6.0 (3.0, 12.5)		0.198 ^d^	0.843
BMI (%)			0.139	4.709 ^b^	0.194
Low weight	11 (9.1)	13 (10.7)			
Normal	59 (48.8)	73 (60.3)			
Overweight	41 (33.8)	29 (24.0)			
Obesity	10 (8.3)	6 (5.0)			
HAMD anxiety subscale	5.8 ± 2.2	5.8 ± 1.8	0.000	−0.129 ^a^	0.897
HAMD weight subscale	1.0 (0.0, 2.0)	0.0 (0.0, 1.0)		−1.757 ^d^	0.080
HAMD cognition subscale	3.0 ± 1.9	3.0 ± 1.6	0.000	−0.073 ^a^	0.942
HAMD retard subscale	6.8 ± 1.7	6.7 ± 1.7	0.059	−0.675 ^a^	0.500
HAMA somatic anxiety	13.4 ± 4.1	13.3 ± 3.6	0.026	− 0.133 ^a^	0.894
HAMA psychological anxiety	6.9 ± 4.3	7.2 ± 3.8	0.066	0.615 ^a^	0.539

*G1* Group 1, patients with severe sleep disturbance, *G2* Group 2, without severe sleep disturbance

^a^ Independent sample t-test; ^b^ chi-square test; ^c^ Fisher’s exact test; ^d^ nonparametric test

Before matching, compared with the patients in Group 2 (no severe sleep disturbance), chi-square analysis revealed that patients in Group 1 (severe sleep disturbance) had more impairment in executive functioning (32.2% vs 20.6%; *P* = 0.024, *q* = 0.036), and executive shifting (55.4% vs 40.0%; *P* = 0.009, *q* = 0.027), and executive inhibition (35.5% vs 23.4%, *P* = 0.036), respectively. After propensity score matching, for Group 1, the odds ratio for having a greater degree of cognitive impairment in executive function was 1.922 (95% CI: 1.068, 3.459, *P* = 0.029, *q* = 0.044), and that for executive shifting was 2.023 (95% CI: 1.211, 3.379, *P* = 0.007, *q* = 0.021) (Tables 3 and 4).

**Table 3:** Cognitive impairment in patients before matching

Cognitive domains	G1 (n, %)	G2 (n, %)	Chi-square	OR	95%CI	*P*	*q*
Executive function	39 (32.2)	36 (20.6)	5.141	1.836	1.082, 3.116	0.024	0.036
Executive shifting	67 (55.4)	70 (40.0)	6.799	1.816	1.164, 2.975	0.009	0.027
Executive inhibition	43 (35.5)	41 (23.4)	5.160	1.802	1.081, 3.003	0.024	0.036

*G1* Group 1, patients with severe sleep disturbance (HAMD-17 sleep score > 4), *G2* Group 2, patients without severe sleep disturbance (HAMD-17 sleep score ≤ 4)

**Table 4:** Cognitive impairment in patients after matching

Cognitive domains	G1 (n, %)	G2 (n, %)	Chi-square	OR	95%CI	*P*	*q*
Executive function	39 (32.2)	23 (19.0)	5.551	1.922	1.068, 3.459	0.029	0.044
Executive shifting	67 (55.4)	48 (39.7)	5.982	2.023	1.211, 3.379	0.007	0.021
Executive inhibition	43 (35.5)	27 (22.3)	5.146	1.749	1.000, 3.060	0.050	0.050

*G1* Group 1, patients with severe sleep disturbance (HAMD-17 sleep score > 4), *G2* Group 2, patients without severe sleep disturbance (HAMD-17 sleep score ≤ 4)

### Subgroup and sensitivity analyses

We analyzed the effects of severe sleep disturbance stratified by age, sex, and depression severity on executive function domain.

We evaluated the effects of severe sleep disturbance on executive function in different age group. (Table S1–3). In patients aged < 30 years, sleep disturbance had influence on executive shifting (OR = 13.714, 95% CI: 2.739, 68.678, *p* = 0.001, *q* = 0.003). In patients aged ≥30 and < 45, effect was observed on executive function (OR = 3.450, 95%CI: 1.367, 8.704, *p* = 0.009, *q* = 0.027). Such an effect was not obvious after correction of multiple tests in executive inhibition (OR = 2.568, 95% CI: 1.039, 6.344, *p* = 0.041, *q* = 0.061). The effects of sleep disturbance on executive function were not observed in patients aged> 45.

We performed subgroup analyses of the effects of severe sleep disturbance on sex.(Table S4–5). For male, the effect was not observed on executive function. In women, the effect was obvious on executive shifting (OR = 2.358, 95% CI: 1.274, 4.365, *p* = 0.006, *q* = 0.018), which was not obvious in executive function after correcting of multiple tests (OR = 2.135, 95% CI: 1.031, 4.420, *p* = 0.041, *q* = 0.061).

Since the severe sleep disturbance was associated with a more serious MDD, we assessed the effect on different MDD severity groups by HAMD-17 scores after the sleep-subscale score was removed. The MDD severity was split into two degrees according to the median score (17) of the remained HAMD score. Executive shifting (OR = 2.777, 95% CI: 1.386,5.564, *P* = 0.004, *q* = 0.006) and inhibition (OR = 3.556, 95% CI: 1.507,8.386, *P* = 0.003, *q* = 0.009) were obviously influenced in patients with HAMD≥17, while the effect was not observed in patients with HAMD < 17.

## Discussion

In this study, we evaluated the effects of severe sleep disturbance on cognitive functioning in treatment-naïve patients with first-episode MDD and found a link between severe sleep disturbance and impaired executive functioning. We found significant differences in stratification by sex, age, and depression severity between Groups 1 and 2.

The baseline characteristics of the 2 groups had significant heterogeneity, so we performed propensity score matching to reduce bias, which reduced the sample size to 242 participants and enabled us to perform between-group comparisons, though the statistical power was decreased because the sample was somewhat smaller. There was a difference in the effect size of age between the 2 groups, though we had already considered the effect of age when we calculated the global deficit scores.

In our study, severe sleep disturbance was related to impaired executive function and executive shifting in patients with MDD, while such an effect was not obvious on executive inhibition. The result was in line with a previous study that MDD patients with poorer sleep quality contribute to impaired executive function [18]. Though no statistically significant difference in executive inhibition was observed between severe sleep disturbance group and no severe sleep disturbance group, statistical significance was observed through subgroup analysis among patients with more serious depression.

We extended the previous research on relationship between insomnia and impaired executive function in adult by assessing different dimensions of executive function in MDD patients [17]. The different effects of severe sleep disturbance on executive dimensions suggested distinct responsible brain region and organization in different tasks, though the dimensions of executive inhibition and shifting were co-related with each other [36, 37]. Few study explored the mechanism of which sleep disturbance is associated with a poor executive function in MDD patients. However, functional magnetic resonance (fMRI) studies about executive inhibition in unmedicated first-episode MDD patients revealed decreased prefrontal function and disrupted functional connectivity of prefrontal cortex and the left cerebellum at the rest state [38], which supported our hypothesis that sleep disturbance in MDD is related to a deactivated prefrontal cortex and less connection of corti-subcorti. Moreover, patients with insomnia showed less functional connective variability between the anterior salience network and the executive-inhibition network, rather than less network functional connectivity strength [39]. The impaired executive function in MDD was related to not only distinct alteration of brain regions, but also the functional network. The alteration of fMRI of sleep disturbance on executive inhibition in MDD was differed from that on insomnia, which can explain the insignificant result found on executive inhibition. Moreover, further study of distinct alteration of sleep in MDD is essential for a better understanding of how executive impairment is developed.

We stratified study participants by age, sex, and severity of MDD and analyzed the effects of sleep disturbances in executive function. Our results showed that patients who fell into discrete categories of age, sex, and depression severity were more impaired by severe sleep disturbance. Specifically, patients younger than 30 years were more likely to experience cognitive impairment in executive shifting. Several analyses did not reveal the effect of age on executive function in MDD patients [8], while the executive flexibility matured at early adulthood and decreased along with age [40]. Therefore, sleep disturbances were related to executive flexibility more in younger MDD patients due to the delayed development during the key period of executive flexibility. Furthermore, executive inhibition matured at the age of adolescence, so it was less impacted by sleep disturbances at adulthood. We found that patients between the ages of 30 and 45 were more likely to have deficits in executive functioning. The general executive function peaked at the third decade of life span, and disturbed sleep seemed to influence such a process [41].

We did observe a difference by sex. Severe sleep disturbance were more likely to affect women in executive shifting. Sex difference existed in cognitive processes and females were more influenced by sleep disturbance on cognitive function, which has been reported by several previous studies [42, 43]. Orexin may modulate executive shifting and sleep in female mice, which can explain the sex difference found in our study [44]. A recent meta-analysis found an insignificant influence of sex difference on executive inhibition, which is in line with our founding [45]. As PFC was affected by oestrogen and susceptible to fluctuations across endogenous hormone cycle [46], the effects of hormone level on distinct executive dimensions cannot be ruled out.

Cognitive impairment has also been reported to be related to MDD severity. Patients with severe depression are more likely to show impairment in executive inhibition and executive shifting. The severe sleep disturbance was related to a more serious depression, which could be “double hit” on brain function and related to poorer executive shifting and inhibition. However, executive impairment was considered as a trait-like feature that is not secondary to MDD severity and often persisted after clinical remission [6]. Therefore, the proportion of general executive function impairment was stable in patients with different level of MDD severity. Here we evaluated executive function impairment in a more strict way and calculated GDS according to stroop color word test and color line II; the rate of executive impairment is lower than that of executive-inhibition and executive-shift in different MDD severity categories.

Our study had some limitations. The cross-sectional study data we accessed had a weak cause-and-effect relationship. Future longitudinal studies will be essential in evaluating the link between sleep disturbance remission and cognitive impairment in MDD patients. Moreover, further neurobiological mechanism study is necessary to explore the underlining association between sleep disturbance and executive function in MDD patients. Our sample population was restricted to Chinese adults aged 18 to 65 years, so our results may not be generalizable to other populations. Also, the ages of the participants in our study varied so widely that a between-group imbalance in age ranges remained after propensity score matching. Meanwhile, we did not include work memory assessment in this study. Finally, we used the HAMD sleep subscale to estimate severe sleep disturbance rather than objective measurement such as polysomnography. More specific measurement tools will be necessary to evaluate the effects of the stages of sleep in patients with MDD.

## Conclusions

We explored the link between symptoms of sleep disturbance and executive function impairment in first-episode, treatment naïve patients with MDD, and stratified the effect of sleep disturbance symptoms by age, sex, and depression severity in different domains. To evaluate the presence and degree of cognitive impairment, we used a battery of comprehensive, cognitive measurement tests, which have proved sensitive in identifying impaired cognition in mental disorders.

Our results indicate that severe sleep disturbance is associated with impaired executive functioning in first-episode, treatment-naïve patients with MDD. Certain patients, including women,age < 45, with severe depression, may be more likely to exhibit the symptoms of executive impairment. Severe sleep disturbance can be a marker of executive function impairment and can aid in recognizing executive impairment in patients with MDD. Moreover, severe sleep disturbance can be a potential modifiable factor to improve executive function, as well as an effective measurement to improve cognition for sleep symptoms management that should be enforced at initial treatment of first-episode MDD.

## Supplementary Information

**Additional file 1.**

## Data Availability

The dataset analyzed during the current study are available from the corresponding author on reasonable request.

## Abbreviations

MDD: Major depressive disorder
MINI: Mini-International Neuropsychiatric Interview
HAMD-17: 17-item Hamilton Rating Scale for Depression
HAMA: Hamilton Anxiety Rating Scale
CTT: Color Trial Test
SCWT: Stroop Color Word Test
MATRICS: The Measurement and Treatment Research to Improve Cognition in Schizophrenia
MCCB: MATRICS Consensus Cognitive Battery
GDS: Global deficit score
BMI: Body mass index
FBG: Fasting blood glucose
TC: Total cholesterol
TG: Triglycerides
LDL: Low-density lipoprotein
CTQ: Childhood trauma questionnaire

## References

[1] Zuckerman H, Pan Z, Park C, Brietzke E, Musial N, Shariq AS, Iacobucci M, Yim SJ, Lui L, Rong C (2018). Recognition and treatment of cognitive dysfunction in major depressive disorder. Front Psychiatry.

[2] Clark M, DiBenedetti D, Perez V (2016). Cognitive dysfunction and work productivity in major depressive disorder. Expert Rev Pharmacoecon Outcomes Res.

[3] Carvalho AF, Miskowiak KK, Hyphantis TN, Kohler CA, Alves GS, Bortolato B, Sales MG, Machado-Vieira R, Berk M, McIntyre RS (2014). Cognitive dysfunction in depression - pathophysiology and novel targets. CNS Neurol Disord Drug Targets.

[4] Conradi HJ, Ormel J, de Jonge P (2011). Presence of individual (residual) symptoms during depressive episodes and periods of remission: a 3-year prospective study. Psychol Med.

[5] Ahern E, Semkovska M (2017). Cognitive functioning in the first-episode of major depressive disorder: a systematic review and meta-analysis. Neuropsychology.

[6] Reppermund S, Ising M, Lucae S, Zihl J (2009). Cognitive impairment in unipolar depression is persistent and non-specific: further evidence for the final common pathway disorder hypothesis. Psychol Med.

[7] Simons CJ, Jacobs N, Derom C, Thiery E, Jolles J, van Os J, Krabbendam L (2009). Cognition as predictor of current and follow-up depressive symptoms in the general population. Acta Psychiatr Scand.

[8] Snyder HR (2013). Major depressive disorder is associated with broad impairments on neuropsychological measures of executive function: a meta-analysis and review. Psychol Bull.

[9] Clark L, Chamberlain SR, Sahakian BJ (2009). Neurocognitive mechanisms in depression: implications for treatment. Annu Rev Neurosci.

[10] Boland EM, Vittengl JR, Clark LA, Thase ME, Jarrett RB (2020). Is sleep disturbance linked to short- and long-term outcomes following treatments for recurrent depression?. J Affect Disord.

[11] Goldstone A, Javitz HS, Claudatos SA, Buysse DJ, Hasler BP, de Zambotti M, Clark DB, Franzen PL, Prouty DE, Colrain IM, Baker FC (2020). Sleep disturbance predicts depression symptoms in early adolescence: initial findings from the adolescent brain cognitive development study. J Adolesc Health.

[12] Stickley A, Leinsalu M, DeVylder JE, Inoue Y, Koyanagi A (2019). Sleep problems and depression among 237 023 community-dwelling adults in 46 low- and middle-income countries. Sci Rep.

[13] Steiger A, Kimura M (2010). Wake and sleep EEG provide biomarkers in depression. J Psychiatr Res.

[14] Maquet P, Ruby P, Maudoux A, Albouy G, Sterpenich V, Dang-Vu T, Desseilles M, Boly M, Perrin F, Peigneux P (2005). Human cognition during REM sleep and the activity profile within frontal and parietal cortices: a reappraisal of functional neuroimaging data. Prog Brain Res.

[15] Ling A, Lim ML, Gwee X, Ho RC, Collinson SL, Ng TP (2016). Insomnia and daytime neuropsychological test performance in older adults. Sleep Med.

[16] Grau-Rivera O, Operto G, Falcon C, Sanchez-Benavides G, Cacciaglia R, Brugulat-Serrat A, Gramunt N, Salvado G, Suarez-Calvet M, Minguillon C (2020). Association between insomnia and cognitive performance, gray matter volume, and white matter microstructure in cognitively unimpaired adults. Alzheimers Res Ther.

[17] Wardle-Pinkston S, Slavish DC, Taylor DJ (2019). Insomnia and cognitive performance: a systematic review and meta-analysis. Sleep Med Rev.

[18] Cabanel N, Schmidt AM, Fockenberg S, Bruckmann KF, Haag A, Muller MJ, Kundermann B (2019). Evening preference and poor sleep independently affect attentional-executive functions in patients with depression. Psychiatry Res.

[19] Cockshell SJ, Mathias JL (2013). Cognitive deficits in chronic fatigue syndrome and their relationship to psychological status, symptomatology, and everyday functioning. Neuropsychology.

[20] Thomas AJ, Gallagher P, Robinson LJ, Porter RJ, Young AH, Ferrier IN, O'Brien JT (2009). A comparison of neurocognitive impairment in younger and older adults with major depression. Psychol Med.

[21] van Agtmaal M, Houben A, Pouwer F, Stehouwer C, Schram MT (2017). Association of microvascular dysfunction with late-life depression: a systematic review and meta-analysis. Jama Psychiat.

[22] Lemos-Miller A, Kearney CA (2006). Depression and ethnicity as intermediary variables among dissociation, trauma-related cognitions, and PTSD symptomatology in youths. J Nerv Ment Dis.

[23] Dannehl K, Rief W, Euteneuer F (2017). Childhood adversity and cognitive functioning in patients with major depression. Child Abuse Negl.

[24] Withall A, Harris LM, Cumming SR (2010). A longitudinal study of cognitive function in melancholic and non-melancholic subtypes of major depressive disorder. J Affect Disord.

[25] Zimmerman ME, Bigal ME, Katz MJ, Brickman AM, Lipton RB (2012). Sleep onset/maintenance difficulties and cognitive function in nondemented older adults: the role of cognitive reserve. J Int Neuropsychol Soc.

[26] Rosenstrom T, Jokela M, Puttonen S, Hintsanen M, Pulkki-Raback L, Viikari JS, Raitakari OT, Keltikangas-Jarvinen L (2012). Pairwise measures of causal direction in the epidemiology of sleep problems and depression. PLoS One.

[27] Bagherzadeh-Azbari S, Khazaie H, Zarei M, Spiegelhalder K, Walter M, Leerssen J, Van Someren E, Sepehry AA, Tahmasian M (2019). Neuroimaging insights into the link between depression and insomnia: a systematic review. J Affect Disord.

[28] Hamilton M (1960). A rating scale for depression. J Neurol Neurosurg Psychiatry.

[29] Lv X, Si T, Wang G, Wang H, Liu Q, Hu C, Wang J, Su Y, Huang Y, Jiang H, Yu X (2016). The establishment of the objective diagnostic markers and personalized medical intervention in patients with major depressive disorder: rationale and protocol. BMC Psychiatry.

[30] Sheehan DV, Lecrubier Y, Sheehan KH, Amorim P, Janavs J, Weiller E, Hergueta T, Baker R, Dunbar GC (1998). The Mini-International Neuropsychiatric Interview (M.I.N.I.): the development and validation of a structured diagnostic psychiatric interview for DSM-IV and ICD-10. J Clin Psychiatry.

[31] Wang XD, Wang XL, Ma H (1999). Handbook of mental health assessment scale.

[32] Hamilton M (1959). The assessment of anxiety states by rating. Br J Med Psychol.

[33] Yu X (2014). Handbook of MATRICS consensus cognitive battery Chinese norm.

[34] Carey CL, Woods SP, Gonzalez R, Conover E, Marcotte TD, Grant I, Heaton RK, HNRC G (2004). Predictive validity of global deficit scores in detecting neuropsychological impairment in HIV infection. J Clin Exp Neuropsyc.

[35] Bernstein DP, Fink L, Handelsman L, Foote J, Lovejoy M, Wenzel K, Sapareto E, Ruggiero J (1994). Initial reliability and validity of a new retrospective measure of child abuse and neglect. Am J Psychiatry.

[36] Sylvester CY, Wager TD, Lacey SC, Hernandez L, Nichols TE, Smith EE, Jonides J (2003). Switching attention and resolving interference: fMRI measures of executive functions. Neuropsychologia.

[37] Collette F, Van der Linden M, Laureys S, Delfiore G, Degueldre C, Luxen A, Salmon E (2005). Exploring the unity and diversity of the neural substrates of executive functioning. Hum Brain Mapp.

[38] Shi Y, Li J, Feng Z, Xie H, Duan J, Chen F, Yang H (2020). Abnormal functional connectivity strength in first-episode, drug-naïve adult patients with major depressive disorder. Prog Neuro-Psychopharmacol Biol Psychiatry.

[39] Wei Y, Leerssen J, Wassing R, Stoffers D, Perrier J, Van Someren E (2020). Reduced dynamic functional connectivity between salience and executive brain networks in insomnia disorder. J Sleep Res.

[40] Igazság B, Demetrovics Z, Cserjési R (2019). The developmental trajectory of executive functions and their stress sensitivity in adolescence. Psychiatr Hung.

[41] Dias BF, Rezende LO, Malloy-Diniz LF, Paula JJ (2018). Relationship between visuospatial episodic memory, processing speed and executive function: are they stable over a lifespan?. Arq Neuropsiquiatr.

[42] LaClair M, Febo M, Nephew B, Gervais NJ, Poirier G, Workman K, Chumachenko S, Payne L, Moore MC, King JA et al. Sex differences in cognitive flexibility and resting brain networks in middle-aged marmosets. eNeuro. 2019;6(4):119–54. 10.1523/ENEURO.0154-19.2019 PMC6658914 31262949

[43] Hajali V, Andersen ML, Negah SS, Sheibani V (2019). Sex differences in sleep and sleep loss-induced cognitive deficits: the influence of gonadal hormones. Horm Behav.

[44] Durairaja A, Fendt M. Orexin deficiency modulates cognitive flexibility in a sex-dependent manner. Genes Brain Behav. 2021;20(3):e12707. 10.1111/gbb.12707 33070452

[45] Gaillard A, Fehring DJ, Rossell SL. A systematic review and meta-analysis of behavioural sex differences in executive control. Eur J Neurosci. 2021;53(2):519–42. 10.1111/ejn.14946 32844505

[46] Sun J, Walker AJ, Dean B, van den Buuse M, Gogos A (2016). Progesterone: the neglected hormone in schizophrenia? A focus on progesterone-dopamine interactions. Psychoneuroendocrino.

