# Supplementary material for: Severe sleep disturbance is associated with executive function impairment in patients with first-episode, treatment-naïve major depressive disorders
Source: BMC Psychiatry. 2021 Apr 19;21:198. doi: 10.1186/s12888-021-03194-2 (PMC8054425; doi:10.1186/s12888-021-03194-2)
Supplement: Supplementary file 1 — Additional file 1. [file 12888_2021_3194_MOESM1_ESM.docx]

Table S1 sleep disturbance and executive function in patients age <30

|  | **G1 (n, %)** | **G2 (n, %)** | **OR** | **95%CI** | ***P*** | ***q*** |
| --- | --- | --- | --- | --- | --- | --- |
| Executive function | 8 (44.4) | 9 (23.7) | 2.578 | 0.782, 8.500 | 0.120 | 0.180 |
| Executive shifting | 16 (88.9) | 14 (36.8) | 13.714 | 2.739, 68.678 | 0.001 | 0.003 |
| Executive inhibition | 10 (55.6) | 13 (34.2) | 2.404 | 0.764, 7.562 | 0.134 | 0.134 |

G1 = Group 1, patients with severe sleep disturbance ; G2 = Group 2, patients without severe sleep disturbance .

Table S2 sleep disturbance and executive function in patients age ≥30, <45

|  | **G1 (n, %)** | **G2 (n, %)** | **OR** | **95%CI** | ***P*** | ***q*** |
| --- | --- | --- | --- | --- | --- | --- |
| Executive function | 21 (37.5) | 8 (14.8) | 3.450 | 1.367, 8.704 | 0.009 | 0.027 |
| Executive shifting | 25 (44.6) | 17 (31.5) | 1.755 | 0.805, 3.826 | 0.157 | 0.157 |
| Executive inhibition | 19 (33.9) | 9 (16.7) | 2.568 | 1.039, 6.344 | 0.041 | 0.061 |

G1 = Group 1, patients with severe sleep disturbance ; G2 = Group 2, patients without severe sleep disturbance .

Table S3 sleep disturbance and executive function in patients age ≥45

|  | **G1 (n, %)** | **G2 (n, %)** | **OR** | **95%CI** | ***P*** |
| --- | --- | --- | --- | --- | --- |
| Executive function | 10 (21.3) | 7 (24.1) | 0.849 | 0.283, 2.553 | 0.771 |
| Executive shifting | 26 (55.3) | 15 (51.7) | 1.156 | 0.457, 2.923 | 0.760 |
| Executive inhibition | 14 (29.8) | 7 (24.1) | 1.333 | 0.464, 3.831 | 0.693 |

G1 = Group 1, patients with severe sleep disturbance ; G2 = Group 2, patients without severe sleep disturbance .

Table S4 sleep disturbance and executive function in male patients

|  | **G1 (n, %)** | **G2 (n, %)** | **OR** | **95%CI** | ***P*** |
| --- | --- | --- | --- | --- | --- |
| Executive function | 14 (35.9) | 9 (27.3) | 1.493 | 0.545, 4.090 | 0.435 |
| Executive shifting | 18 (46.2) | 12 (36.4) | 1.500 | 0.581, 3.872 | 0.402 |
| Executive inhibition | 23 (59.0) | 14 (42.4) | 1.951 | 0.762, 4.994 | 0.164 |

G1 = Group 1, patients with severe sleep disturbance ; G2 = Group 2, patients without severe sleep disturbance .

Table S5 sleep disturbance and executive function in female patients

|  | **G1 (n, %)** | **G2 (n, %)** | **OR** | **95%CI** | ***P*** | ***q*** |
| --- | --- | --- | --- | --- | --- | --- |
| Executive function | 25 (30.5) | 15 (17.0) | 2.135 | 1.031, 4.420 | 0.041 | 0.061 |
| Executive shifting | 49 (59.8) | 34 (38.6) | 2.358 | 1.274, 4.365 | 0.006 | 0.018 |
| Executive inhibition | 20 (24.4) | 15 (17.0) | 1.570 | 0.741, 3.324 | 0.239 | 0.239 |

G1 = Group 1, patients with severe sleep disturbance ; G2 = Group 2, patients without severe sleep disturbance .

Table S6 sleep disturbance and executive function in patients remained HAMD-17 ≥17

|  | **G1 (n, %)** | **G2 (n, %)** | **OR** | **95%CI** | ***P*** | ***q*** |
| --- | --- | --- | --- | --- | --- | --- |
| Executive function | 20(29.0%) | 12(17.4%) | 1.939 | 0.861,4.363 | 0.110 | 0.110 |
| Executive shifting | 39(56.5%) | 22(31.9%) | 2.777 | 1.386,5.564 | 0.004 | 0.006 |
| Executive inhibition | 24(34.8%) | 9(13.0%) | 3.556 | 1.507,8.386 | 0.003 | 0.009 |

Remained HAMD-17 score was calculated by sleep subscale removed from total HAMD-17. The MDD severity was split into two degrees according to the median score (median=17) of remained HAMD score.

G1 = Group 1, patients with severe sleep disturbance ; G2 = Group 2, patients without severe sleep disturbance .

Table S7 sleep disturbance and executive function in patients remained HAMD-17 <17

|  | **G1 (n, %)** | **G2 (n, %)** | **OR** | **95%CI** | ***P*** |
| --- | --- | --- | --- | --- | --- |
| Executive function | 19(36.5) | 12(23.1) | 1.919 | 0.814,4.523 | 0.136 |
| Executive shifting | 28(53.8) | 24(46.2) | 0.433 | 0.630,1.361 | 0.433 |
| Executive inhibition | 19(36.5) | 20(38.5) | 0.840 | 0.416,2.038 | 0.921 |

Remained HAMD-17 score was calculated by sleep subscale removed from total HAMD-17. The MDD severity was split into two degrees according to the median score (median=17) of remained HAMD score.

G1 = Group 1, patients with severe sleep disturbance ; G2 = Group 2, patients without severe sleep disturbance .
